# Handgrip Strength Relative to Estimated Skeletal Muscle Mass and Threshold-Defined Physical Performance in Biochemically Controlled Acromegaly: A Cross-Sectional Study

**DOI:** 10.3390/jcm15197412

**Published:** 2026-09-24

**Authors:** Ayşe Merve Ok Kurt, Ümmü Mutlu, Fatih Bektaş, Hulya Hacisahinogullari, Gülşah Yenidünya Yalın, Tuğba Erdoğan, Gülistan Bahat, Nurdan Gul, Ayşe Kubat Üzüm, Ferihan Aral, Sema Yarman, Özlem Soyluk Selçukbiricik

**Affiliations:** 1 Division of Endocrinology and Metabolism, Department of Internal Medicine, Istanbul Faculty of Medicine, Istanbul University, 34093 Istanbul, Türkiye; ukorkmaz18@gmail.com (Ü.M.); drfatihbektas@gmail.com (F.B.); mercandogru@hotmail.com (H.H.); gulsah_y@hotmail.com (G.Y.Y.); nurdan.gul@istanbul.edu.tr (N.G.); ayse.kubat@istanbul.edu.tr (A.K.Ü.); ferihanar@yahoo.com (F.A.); sema.yarman@gmail.com (S.Y.); drozlem76@hotmail.com (Ö.S.S.); 2 Department of Endocrinology and Metabolism, Kocaeli City Hospital, 41060 Kocaeli, Türkiye; 3 Department of Endocrinology and Metabolism, Bağcılar Training and Research Hospital, 34200 Istanbul, Türkiye; 4 Division of Geriatrics, Department of Internal Medicine, Istanbul Faculty of Medicine, Istanbul University, 34093 Istanbul, Türkiye; tubaobekli@hotmail.com (T.E.); gbahatozturk@yahoo.com (G.B.)

**Keywords:** acromegaly, handgrip strength, skeletal muscle mass, physical performance, gait speed, chair stand, bioelectrical impedance analysis

## Abstract

**Background/Objectives:** Muscle quantity, strength, and performance may not change in parallel in acromegaly. We compared absolute handgrip strength, estimated skeletal muscle mass (SMM), and handgrip strength relative to SMM (analyzed continuously) by threshold-defined gait and chair-stand impairment. **Methods:** This cross-sectional study of 76 adults with biochemically controlled acromegaly estimated SMM as 0.566 × bioimpedance-derived fat-free mass; gait (≤0.8 m/s) and chair-stand (>15 s) results were binary. Six full-cohort exact permutation comparisons (Holm-adjusted) formed the primary analysis. As no men met either threshold, a post hoc women-only sensitivity analysis was added. **Results:** Gait and chair-stand impairment occurred in four and three participants, respectively (five overall, all women with diabetes). In full-cohort comparisons, gait impairment was associated with lower absolute (Holm-adjusted *p* = 0.0035) and relative (*p* = 0.0061) handgrip strength, and chair-stand impairment with lower relative handgrip strength (*p* = 0.0012); estimated SMM did not differ clearly. In the women-only sensitivity analysis, median absolute and relative handgrip strength with versus without gait impairment were 26.65 vs. 32.15 kg and 0.918 vs. 1.142 kg/kg (both Holm-adjusted *p* = 0.0486). Median estimated SMM was numerically higher in women with chair-stand impairment (31.41 vs. 27.05 kg), so lower relative handgrip strength may partly reflect a larger denominator. In women with diabetes, no exploratory comparison remained below 0.05 after Holm adjustment. **Conclusions:** Lower relative handgrip strength accompanied impairment without a clear reduction in estimated SMM, but few events, all in women with diabetes, limit interpretation. These hypothesis-generating findings do not establish an acromegaly-specific muscle phenotype.

## 1. Introduction

Acromegaly is characterized by chronic growth hormone (GH) and insulin-like growth factor 1 (IGF-1) excess and by multisystem complications that may persist despite biochemical control [1,2]. Active disease is associated with characteristic changes in body composition, including increased lean tissue and reduced adiposity, and these compartments change after treatment [3]. However, body composition alone may not fully reflect muscle strength or physical performance in patients with controlled acromegaly [4]. This provides a rationale for considering muscle quantity and function as related but distinct aspects of musculoskeletal health.

Earlier studies reported reduced grip strength and endurance at diagnosis, with greater proximal muscle fatigability observed during long-term follow-up [5], as well as intramuscular fatty infiltration associated with poorer physical function in controlled acromegaly [6]. Other studies have described abnormalities in lower-extremity muscle and tendon structure [7], forearm muscle mechanical properties assessed by shear-wave elastography [8], and osteosarcopenia with reduced muscle quality [9]. More recent work has extended this picture: studies published in 2025 and 2026 have reported heterogeneous findings for sarcopenic obesity, frailty, and physical function using body-composition and functional assessments [10,11]. Taken together, these findings suggest that muscle quantity, strength, composition, and physical performance should be considered separately rather than assumed to change in parallel.

Handgrip strength relative to estimated SMM provides a simple way to express force in relation to estimated muscle quantity. However, it should not be interpreted as a direct measure of muscle composition or as a validated index of muscle quality [12,13]. Because ratio measures are influenced by the denominator and by body size, findings based on handgrip/SMM should also be examined using an alternative model in which absolute handgrip strength is the outcome and estimated SMM is entered as a covariate.

The present study aimed to examine whether absolute handgrip strength, estimated SMM, and handgrip strength relative to estimated SMM differed according to separately defined gait-speed and chair-stand impairment in adults with biochemically controlled acromegaly. Relative handgrip strength was included in the original analytic framework and was analyzed as a continuous measure without a diagnostic cutoff. Exploratory analyses also examined clinical correlates of relative handgrip strength and compared these findings with an absolute-handgrip model that included estimated SMM as a covariate.

## 2. Materials and Methods

### 2.1. Study Design and Participants

This single-center cross-sectional study screened consecutive adults with acromegaly evaluated at a tertiary endocrinology center between July and December 2022. Diagnosis was based on characteristic clinical findings, elevated age- and sex-adjusted IGF-1, inadequate GH suppression during a 75 g oral glucose tolerance test when available, and pituitary imaging. Biochemical control was defined as an IGF-1 concentration within the age- and sex-specific reference interval for at least six consecutive months, whether achieved after surgery, during medical therapy, or following radiotherapy. Control was confirmed by serial age- and sex-adjusted IGF-1 measurements during this period; a stable treatment regimen was not required, provided IGF-1 remained within the reference interval. Serial GH or OGTT measurements were not uniformly available throughout follow-up; therefore, age- and sex-adjusted IGF-1 was used as the longitudinal criterion for biochemical control. Accordingly, biochemical control was classified on the basis of IGF-1, without a separate category for GH–IGF-1 discordance. At the study assessment, IGF-1 was measured using a SNIBE assay (Shenzhen New Industries Biomedical Engineering Co., Ltd., Shenzhen, China) and interpreted according to the laboratory’s age- and sex-specific reference interval. Assay platforms used for historical baseline IGF-1 measurements were not consistently retrievable from the medical records.

Patients with active acromegaly were excluded. Other exclusions were known neuromuscular disease, fibrous dysplasia including McCune–Albright syndrome, X-linked acrogigantism (X-LAG, excluded because childhood-onset overgrowth had produced extreme stature and severe secondary arthropathy that limited mobility independent of muscle strength and fell outside the height range for which the body-composition and handgrip reference equations were validated), clinically significant skeletal deformity, active malignancy, New York Heart Association class III–IV heart failure, end-stage kidney disease, advanced liver failure, severe active infection, pregnancy, and lactation. Of 89 patients assessed for eligibility, 13 were excluded (4 with active acromegaly, 2 with fibrous dysplasia, 1 with McCune–Albright syndrome, 1 with X-LAG, 2 with active malignancy, and 3 who declined participation), leaving 76 participants for analysis (Figure 1). No a priori sample-size calculation was performed; all eligible consenting participants identified through consecutive screening during the study period were included. The study is reported in accordance with the Strengthening the Reporting of Observational Studies in Epidemiology (STROBE) statement.

### 2.2. Clinical and Anthropometric Variables

Demographic, disease, treatment, pituitary-deficiency, and comorbidity data were obtained from medical records and the study visit. The IGF-1 index was calculated as the measured concentration divided by the corresponding age- and sex-specific upper limit of normal. For historical baseline measurements, values were expressed relative to the corresponding age- and sex-specific upper limit of normal because the original assay platform was not consistently documented. Follow-up was the interval from first presentation to the study assessment. Because the available variables did not measure lifetime exposure to GH/IGF-1 excess, the former label ‘duration of active disease’ was not used. Presentation was defined as the first visit to our center, and sustained control was dated from the first normal IGF-1 measurement initiating a continuous period of at least six months within the age- and sex-specific reference interval. Instead, the post-presentation interval to sustained biochemical control was operationalized as follow-up duration minus duration of biochemical control. One negative arithmetic value was set to zero; the final analysis variable contained two zero values. This variable is a center-based interval and not a measure of total disease duration.

Body weight and standing height were measured in light clothing without shoes. Body mass index (BMI) was weight in kilograms divided by height in meters squared; obesity was defined as BMI ≥ 30 kg/m^2^. Waist circumference and other measures of central adiposity were not collected; BMI-defined obesity may therefore not fully characterize adiposity distribution in acromegaly. Diabetes mellitus, hypertension, hyperlipidemia, obstructive sleep apnea, and cardiovascular disease were based on documented diagnoses and/or disease-specific treatment.

### 2.3. Body Composition and Estimated Skeletal Muscle Mass

Body composition was assessed in the morning after an overnight fast and after voiding, using a TANITA BC-420MA bioelectrical impedance analyzer (Tanita Corporation, Tokyo, Japan), a tetrapolar, single-frequency (50 kHz) foot-to-foot system with eight electrodes contacting the plantar surface and heel of each foot. Fat mass, body-fat percentage, and fat-free mass (FFM) were obtained from the device. Estimated total SMM was calculated using the published equation SMM (kg) = 0.566 × FFM (kg) [14]; this constant was derived from comparisons with 24 h urinary creatinine excretion in healthy subjects and has not been specifically validated in acromegaly or in individuals with the degree of obesity observed in this cohort (maximum BMI 77.4 kg/m^2^). Throughout the manuscript, SMM denotes this estimate rather than directly imaged muscle tissue. Because this equation applies a constant multiplicative factor to FFM, handgrip strength normalized to estimated SMM is directly proportional to handgrip strength normalized to FFM (handgrip/SMM = 1.7668 × handgrip/FFM); the SMM-based ratio therefore rescales, rather than adds information beyond, a ratio expressed relative to FFM directly. SMM/BMI was calculated for descriptive purposes; low SMM/BMI was defined as <0.823 in women and <1.049 in men using published Turkish reference values [15]. Turkish reference values were selected because the study population was Turkish, but their use was based on population relevance rather than age matching. These cutoff-based classifications were used only descriptively and did not define the primary performance-group comparisons.

### 2.4. Handgrip Strength and Relative Handgrip Strength

Handgrip strength was measured with a Jamar Plus+ Digital Hand Dynamometer (Performance Health, Warrenville, IL, USA), calibrated shortly before the study began (no recalibration was performed during the approximately 6-month study period), by one of two trained evaluators, after three minutes of rest. Handle position II was used, adjusted to position III when needed to accommodate larger hand size. Participants were seated with the shoulder neutral, elbow flexed at 90°, forearm neutral, and feet supported. Three measurements were obtained from each hand, repeated when the participant indicated readiness rather than at a fixed interval; the maximum value from either hand was analyzed. Low absolute handgrip strength was defined descriptively as <20 kg in women and <35 kg in men using Turkish reference values [16]. Relative handgrip strength was calculated as maximum handgrip strength (kg) divided by estimated SMM (kg) and was analyzed as a continuous measure of strength relative to estimated muscle quantity. It was not classified using unvalidated cutoffs. We use the term ‘relative handgrip strength’ rather than ‘muscle quality index’ because no imaging or histologic measure of muscle composition was available.

### 2.5. Threshold-Defined Physical Performance

Physical performance was assessed at the study visit with a usual-pace 4 m walk, timed with a stopwatch from a static start with no acceleration or deceleration phase, and a five-repetition chair-stand test performed with the arms crossed over the chest using an armless chair 45 cm in height, timed with a stopwatch, with full knee extension required for each repetition to count as valid; no assistive devices were used during either test. All 76 participants completed both tests; none was left incomplete. In accordance with the protocol, gait was classified as impaired at ≤0.8 m/s and chair-stand performance as impaired when completion exceeded 15 s [12]. Each result was classified as normal or impaired by the assessing evaluator at the time of testing; this classification was not independently verified against a separate timing record. The source analysis dataset retained only the resulting normal/impaired classification for each test; individual gait speeds and chair-stand times were not available in the study database. The two tests were therefore analyzed as separate binary grouping variables. Meeting either threshold was reported only as a descriptive summary and was not used as the primary inferential outcome. These thresholds are most often validated in populations enriched for older age; their applicability to the relatively younger age structure of this cohort is addressed in Section 4.

### 2.6. Statistical Analysis

Continuous data are summarized as mean ± standard deviation or median (interquartile range), as appropriate based on descriptive inspection of their distributions; categorical data are presented as n (%). The originally defined primary analysis comprised six full-cohort comparisons: handgrip strength, estimated skeletal muscle mass, and relative handgrip strength across the gait-speed and chair-stand categories. Because the impaired groups contained only four and three participants, respectively, groups were compared with two-sided exact permutation-based Mann–Whitney tests, enumerating all possible group allocations for each comparison (up to 1,282,975 allocations for the four-participant gait comparison). Ties were handled using mid-ranks within the exact enumeration. For each comparison, we additionally report the Hodges–Lehmann estimate of the location shift with an exact 95% confidence interval obtained by inverting the exact Mann–Whitney test; by construction, this interval excludes zero if and only if the corresponding exact test is significant at the matching two-sided alpha level. Effect size was expressed as the rank-biserial correlation, with negative values indicating lower measurements in the impaired group. Holm adjustment controlled the family-wise error rate across the six full-cohort comparisons.

All five participants meeting either threshold were women, and none of the 38 men met either threshold. After this became apparent, an exact permutation test stratified by sex was added as a post hoc sensitivity analysis of the six comparisons: because the male stratum contained no impaired participants, this stratified test reduced mathematically to the women-only comparison, an equivalence confirmed by full enumeration for all six comparisons. Holm adjustment was applied to the six women-only comparisons. This analysis addresses the clustering of impairment by sex but not its clustering among participants with diabetes. As a secondary, model-based check, an asymptotic Van Elteren (stratified Wilcoxon) statistic was also computed. Because all participants meeting either threshold were also diabetic, a further exploratory analysis restricted the comparison group to women with diabetes.

To assess the robustness of the six full-cohort comparisons to individual observations, each analysis was repeated after removing one impaired participant at a time (leave-one-out), with the six-comparison Holm adjustment reapplied within each removal scenario.

Event-based multivariable modeling of performance impairment was not attempted because the number of events was insufficient. Instead, exploratory ordinary least-squares models assessed clinical correlates across the full continuous range of strength. Model A used relative handgrip strength as the outcome. Model B used absolute handgrip strength as the outcome and entered estimated SMM per 5 kg as a covariate, thereby avoiding ratio normalization. Both models included age per 10 years, sex, obesity, diabetes, post-presentation interval to sustained control per five years, previous radiotherapy, and current medical therapy, with HC3 heteroskedasticity-robust standard errors. Collinearity was assessed using variance inflation factors. Model fit was assessed using the Breusch–Pagan test for heteroskedasticity, the Shapiro–Wilk test for residual normality, the Ramsey RESET test for functional form, and squared-term tests for the linearity of continuous predictors. Influence was assessed using Cook’s distance. Both models were repeated after excluding the participant with BMI 77.4 kg/m^2^ and, separately, after excluding the observation with the highest Cook’s distance in each respective model. An allometric sensitivity analysis examined the proportionality assumed by normalizing handgrip strength to estimated SMM, using a log–log model of handgrip strength on estimated SMM to test whether the slope differed from 1, whether the untransformed intercept differed from zero, and whether an estimated SMM-by-sex interaction was present. No missing-value imputation was performed.

Data management and verification used IBM SPSS Statistics for Windows, version 31.0 (IBM Corp., Armonk, NY, USA). Statistical analyses were performed in Python 3.12 using SciPy 1.18 and statsmodels 0.14.6. For the six full-cohort comparisons that constitute the primary analysis, two-sided Holm-adjusted *p*-values < 0.05 were used as the inferential threshold. The post hoc sex-stratified analysis was interpreted as a sensitivity analysis, and the diabetes-restricted analysis and all other analyses as exploratory; for these, Holm-adjusted *p*-values close to 0.05 were not treated as confirmation of a stable association.

### 2.7. Use of Generative AI

ChatGPT (OpenAI, GPT-5.6 Sol, San Francisco, CA, USA) was used solely for English language editing and proofreading. All scientific content was reviewed and approved by the authors.

### 2.8. Ethics

The Istanbul Faculty of Medicine Clinical Research Ethics Committee approved the protocol on 14 June 2022 (approval no. 933080). Written informed consent was obtained from all participants. The study was conducted in accordance with the Declaration of Helsinki.

## 3. Results

### 3.1. Cohort Characteristics

The cohort comprised 38 women and 38 men with a mean age of 48.8 ± 11.0 years (range, 26–72 years). Median follow-up was 118.5 months, median duration of biochemical control was 65.5 months, and the median post-presentation interval to sustained control was 29.0 months. The median estimated SMM was 34.67 kg, the median maximum handgrip strength was 40.25 kg, and the median relative handgrip strength was 1.182 kg/kg. Four participants (5.3%) had impaired gait, three (3.9%) had impaired chair-stand performance, and two met both criteria; five (6.6%) met either threshold. All five were women with diabetes, three also had obesity, and their ages ranged from 41 to 72 years. Because all impairment events occurred among women with diabetes, related analyses are presented in Section 3.2 and Section 3.3. Individual characteristics of these five participants, together with comparative data for women who met neither performance threshold, are provided in Supplementary Table S3 (Panel C). At assessment, 32 participants (42.1%) were receiving a somatostatin receptor ligand (13 octreotide and 19 lanreotide) and 22 (28.9%) a dopamine agonist; 21 participants were receiving both concurrently, so these categories were not mutually exclusive. Overall, 33 participants (43.4%) were receiving current medical therapy. None were receiving pegvisomant or pasireotide, although four participants (5.3%) had previously received pegvisomant. Cohort characteristics are presented in Table 1.

### 3.2. Separate Gait and Chair-Stand Comparisons

The six full-cohort comparisons constitute the originally defined primary analysis. All Hodges–Lehmann confidence intervals reported in this section are unadjusted 95% CIs. In the full-cohort comparisons, participants with impaired gait had lower absolute handgrip strength (rank-biserial r = −0.878, Hodges–Lehmann Δ = −15.8 kg, 95% CI −27.6 to −5.3; Holm-adjusted *p* = 0.0035) and lower relative handgrip strength (rank-biserial r = −0.847, Δ = −0.292 kg/kg, 95% CI −0.477 to −0.127; Holm-adjusted *p* = 0.0061) than those without gait impairment. Estimated SMM showed no clear between-group difference (Δ = −6.88 kg, 95% CI −13.92 to 0.79; Holm-adjusted *p* = 0.2175). None of the four participants with impaired gait met the study’s descriptive threshold for low absolute handgrip strength (<20 kg in women); their individual handgrip values ranged from 22.1 to 27.5 kg. Thus, the between-group difference in handgrip strength did not correspond to low absolute handgrip strength according to the threshold used in this study.

Participants with impaired chair-stand performance had lower relative handgrip strength (rank-biserial r = −0.973, Δ = −0.404 kg/kg, 95% CI −0.637 to −0.209; Holm-adjusted *p* = 0.0012). Estimated SMM showed no clear between-group difference (Δ = 0.45 kg, 95% CI −10.58 to 14.60; Holm-adjusted *p* = 0.9624). The estimated difference in absolute handgrip strength was −13.8 kg (95% CI −29.1 to −1.4), with a Holm-adjusted *p* value of 0.0555. Because all participants meeting either performance threshold were women with diabetes, these full-cohort comparisons are interpreted cautiously. The post hoc sex-stratified sensitivity analysis is presented below, and the diabetes-restricted exploratory analysis is presented in Section 3.3. The individual comparisons are shown in Table 2.

Leave-one-out analyses showed small-sample sensitivity in some comparisons. For gait impairment, the relative-handgrip comparison crossed the 0.05 threshold in two of four removal scenarios, whereas the absolute-handgrip comparison remained below 0.05 throughout. For chair-stand impairment, the relative-handgrip comparison remained below 0.05 after removal of each impaired participant, while the absolute-handgrip comparison fell below 0.05 in one of three removal scenarios. Both estimated-SMM comparisons remained above 0.05 throughout (Supplementary Table S2, Panels A and B). Participant-level distributions are shown in Supplementary Figure S1.

Because no men met either impairment threshold, the exact sex-stratified permutation analysis reduced to the women-only comparison; it was added post hoc as a sensitivity analysis and does not address the clustering of impairment among participants with diabetes. Among women, median absolute and relative handgrip strength were 26.65 vs. 32.15 kg and 0.918 vs. 1.142 kg/kg with versus without gait impairment (Holm-adjusted *p* = 0.0486 for both), whereas there was no clear difference in estimated SMM (Holm-adjusted *p* = 0.7716). Given the very small impaired group and the proximity of the handgrip results to the conventional 0.05 threshold, these values do not provide stronger evidence than the full-cohort comparisons and should not be interpreted as confirming a stable association.

For chair-stand performance among women in this sensitivity analysis, relative handgrip strength was lower in the impaired group (Holm-adjusted *p* = 0.0100), while there was no clear between-group difference in absolute handgrip strength or estimated SMM after adjustment (Holm-adjusted *p* = 0.4634 for both). Median estimated SMM was numerically higher in the chair-stand–impaired group (31.41 vs. 27.05 kg), so the lower relative handgrip strength may partly reflect the larger denominator. Full women-only results are provided in Supplementary Table S1; the equivalence of the exact sex-stratified and women-only permutation analyses, together with a secondary Van Elteren check, is shown in Supplementary Table S3 (Panel B).

### 3.3. Sensitivity and Multivariable Analyses

In an exploratory analysis restricted to women with diabetes (n = 21; 4 with gait impairment and 3 with chair-stand impairment), none of the six Holm-adjusted *p* values was below 0.05 (Supplementary Table S3, Panel A). Given the small subgroup, this analysis cannot distinguish the effects of sex and diabetes clustering from limited statistical precision.

The results of the multivariable models are presented in Table 3. In the relative-handgrip model (n = 76), female sex (B = −0.133, 95% CI −0.233 to −0.033; *p* = 0.009) and obesity (B = −0.136, 95% CI −0.242 to −0.030; *p* = 0.012) were associated with lower relative handgrip strength. In the complementary absolute-handgrip model, each 5 kg higher estimated SMM was associated with 4.73 kg higher handgrip strength (95% CI 1.30 to 8.17; *p* = 0.007), whereas the coefficients for female sex and obesity did not reach the conventional 0.05 threshold. The different coefficient patterns indicate sensitivity to how estimated SMM is incorporated into the analysis. Model diagnostics showed no severe multicollinearity or major departure from linearity, although several influential observations were identified (Supplementary Table S2, Panels C–E). These cohort-level models evaluate variation in handgrip outcomes across the whole cohort and do not adjust the threshold-defined performance-group comparisons described in Section 3.2.

Sensitivity analyses excluding influential observations showed that the relative-handgrip model was generally stable, and the participant with BMI 77.4 kg/m^2^ was not the most influential observation in this model. By contrast, the sex and obesity coefficients in the absolute-handgrip model were more sensitive to which observation was excluded; the positive coefficient for estimated SMM was retained (Supplementary Table S2, Panel D).

Allometric analyses showed no clear departure from proportional scaling between handgrip strength and estimated SMM: the log–log slope was 1.073 and did not differ from 1 (*p* = 0.49), the untransformed intercept did not differ from zero (*p* = 0.73), and there was no evidence of an estimated SMM-by-sex interaction (*p* = 0.44). The corresponding sex-stratified scatterplot is shown in Supplementary Figure S2.

## 4. Discussion

Threshold-defined gait and chair-stand impairment was uncommon in this cohort of adults with biochemically controlled acromegaly, with only five participants meeting either threshold. In the six full-cohort comparisons, gait impairment was accompanied by lower absolute and relative handgrip strength, whereas the clearest difference for chair-stand impairment was in relative handgrip strength. The post hoc women-only sensitivity analysis showed differences in the same direction, but the Holm-adjusted *p* values for gait were only just below 0.05 (0.0486) and should be interpreted cautiously. Estimated SMM did not show a clear between-group difference in either comparison. These findings indicate that the patterns observed for strength and estimated muscle quantity were not identical, but the small number of impairment events, their clustering among women with diabetes, and the absence of a comparison group preclude disease-specific or mechanistic interpretation.

Our findings should be considered in the context of heterogeneous previous observations on muscle structure and function in acromegaly. Füchtbauer et al. reported reduced grip strength and endurance at diagnosis; grip strength improved after biochemical remission, whereas greater proximal muscle fatigability was observed at long-term follow-up [5]. Martel-Duguech et al. found greater thigh intramuscular fat infiltration in patients with controlled acromegaly, together with poorer physical performance, and greater fat infiltration was associated with worse functional performance [6]. Other studies have described abnormalities in lower-extremity muscle and tendon structure [7], altered forearm muscle mechanical properties [8], reduced muscle quality with increased vertebral fat deposition [9], and changes in temporal and masseter muscle composition on MRI [17]. Inojosa et al. reported poorer gait and Timed Up and Go performance despite higher fat-free mass index, without a difference in sarcopenic obesity prevalence [10]. Ugur et al. reported a high prevalence of frailty in acromegaly but a low prevalence of sarcopenia [11]. Rocha et al., using the ESPEN/EASO consensus criteria for sarcopenic obesity [18,19], reported sarcopenic obesity in 16.7% of patients with acromegaly and none of the controls, together with impaired physical performance despite greater lean mass. Differences in disease status, age, adiposity, study populations, body-composition methods, and functional definitions may account for some of these apparently discordant findings.

Relative handgrip strength should be interpreted as force normalized to estimated muscle quantity rather than as a direct measure of muscle tissue quality. Because estimated SMM was calculated as 0.566 × FFM, handgrip/SMM is mathematically equivalent to 1.7668 × handgrip/FFM; the ratio therefore rescales strength relative to FFM rather than providing an independent measure of muscle composition. This issue is particularly relevant to the chair-stand comparison, in which median estimated SMM was numerically higher in women with impaired performance (31.41 vs. 27.05 kg), so the lower relative handgrip value may partly reflect the larger denominator. Changes in the sex and obesity coefficients in the absolute-handgrip model further indicate that interpretation depends on how estimated muscle mass is incorporated into the analysis. Although the allometric analysis showed no clear departure from proportional scaling between handgrip strength and estimated SMM, this does not validate the ratio as a measure of muscle quality. Relative handgrip strength is therefore best regarded here as a descriptive continuous measure rather than a validated measure of muscle quality.

All five participants who met either performance threshold were women with diabetes, and three also had obesity. Because no men met either threshold, the post hoc sex-stratified sensitivity analysis addressed the clustering by sex but not the parallel clustering by diabetes. When analyses were restricted to women with diabetes, none of the six comparisons remained below 0.05 after Holm adjustment, although the subgroup was too small to distinguish confounding from loss of statistical precision. Type 2 diabetes has been associated with altered thigh and regional adipose distribution and greater skeletal muscle adipose infiltration [20,21,22], lower muscle quality on computed tomography [23], accelerated loss of muscle strength [24], and poorer physical performance [25], and may therefore have contributed to the observed pattern. In this small subgroup, the independent contributions of sex, diabetes, obesity, age, and acromegaly-related factors cannot be separated reliably.

Lower-extremity performance in acromegaly may also be influenced by arthropathy, pain, spinal disease, balance, cardiopulmonary status, and habitual physical activity. Acromegalic arthropathy may persist despite biochemical control and can affect mobility through pain and structural joint abnormalities [1,26]. These factors were not systematically recorded in the present study. Handgrip strength assesses an upper-extremity task, whereas gait and chair-stand performance depend on lower-extremity strength as well as joint function, balance, and cardiorespiratory capacity. Notably, none of the four participants with gait impairment met the study’s descriptive cutoff for low absolute handgrip strength, despite the between-group difference in handgrip strength. Thus, differences between handgrip and lower-extremity performance should not be interpreted as evidence of an intrinsic muscle abnormality alone.

Treatment history was heterogeneous: most participants had undergone surgery, 33 were receiving medical therapy at assessment, and 14 had previously received radiotherapy. Previous radiotherapy and current medical therapy did not show clear associations with either relative or absolute handgrip strength in the exploratory models. The absence of clear associations in these models does not exclude treatment effects, because treatment selection reflects prior disease severity and response, current therapy grouped different medical regimens, and the study was cross-sectional. The post-presentation interval to sustained biochemical control also requires cautious interpretation because it is a center-based measure and does not represent lifetime exposure to GH/IGF-1 excess. Radiotherapy is generally reserved for patients with persistent or difficult-to-control disease after other treatments and carries a recognized long-term risk of hypopituitarism [27,28].

This study has several strengths. It used consecutive screening of adults with acromegaly. Body composition was assessed using a defined bioimpedance protocol, and handgrip strength was measured according to a defined protocol. Gait and chair-stand impairment were considered separately, relative handgrip strength was analyzed as a continuous measure without an unvalidated diagnostic cutoff, and the primary comparisons used exact tests with rank-based effect-size estimates and multiplicity adjustment. The analyses addressed clustering by sex and diabetes, the influence of individual observations, and alternative ways of accounting for estimated muscle mass.

The study also has important limitations. It was conducted at a single center and had a cross-sectional design, without an active-acromegaly group or a concurrently recruited non-acromegaly control group; causal and acromegaly-specific conclusions therefore cannot be drawn. Performance impairment was uncommon, with only five participants meeting either threshold, all of whom were women with diabetes. The small number of events limited precision, did not permit multivariable modeling of impairment status, and leave-one-out analyses indicated sensitivity to individual observations in some comparisons. In addition, only binary gait and chair-stand classifications were retained in the source dataset, preventing analysis of the continuous performance measures. The fixed thresholds should also be interpreted cautiously in this relatively young cohort, in which 21.1% of participants were aged ≥60 years, and 7.9% were aged ≥65 years. Estimated SMM was derived from bioimpedance-based FFM using an equation developed in healthy individuals and not specifically validated in acromegaly or extreme obesity. Although measurements were obtained after an overnight fast and voiding, hydration status, recent exercise, alcohol, and caffeine intake were not fully standardized. Direct measures of muscle fat infiltration or architecture were unavailable, and joint pain, arthropathy, physical activity, neuropathy, and other determinants of mobility were not systematically recorded. Finally, relative handgrip strength remains mathematically dependent on estimated SMM, and its interpretation is sensitive to how muscle quantity is incorporated into the analysis.

## 5. Conclusions

Among 76 adults with biochemically controlled acromegaly, gait- and chair-stand impairment was uncommon, and the impaired groups showed lower handgrip strength relative to estimated SMM without a clear reduction in estimated SMM. However, only five participants met either performance threshold, all of whom were women with diabetes. Together with the absence of continuous performance values and concurrent control groups, this clustering limits disease-specific interpretation. Relative handgrip strength should therefore be considered a descriptive continuous measure rather than a validated marker of muscle quality, and the present findings are hypothesis-generating. Future prospective studies should retain continuous performance measurements, include non-acromegaly and active-disease comparison groups, directly assess muscle composition, and account for pain, arthropathy, diabetes, and adiposity. Validation against clinically relevant outcomes would be required before any relative-strength threshold could be proposed for acromegaly.

## Figures and Tables

**Figure 1:**
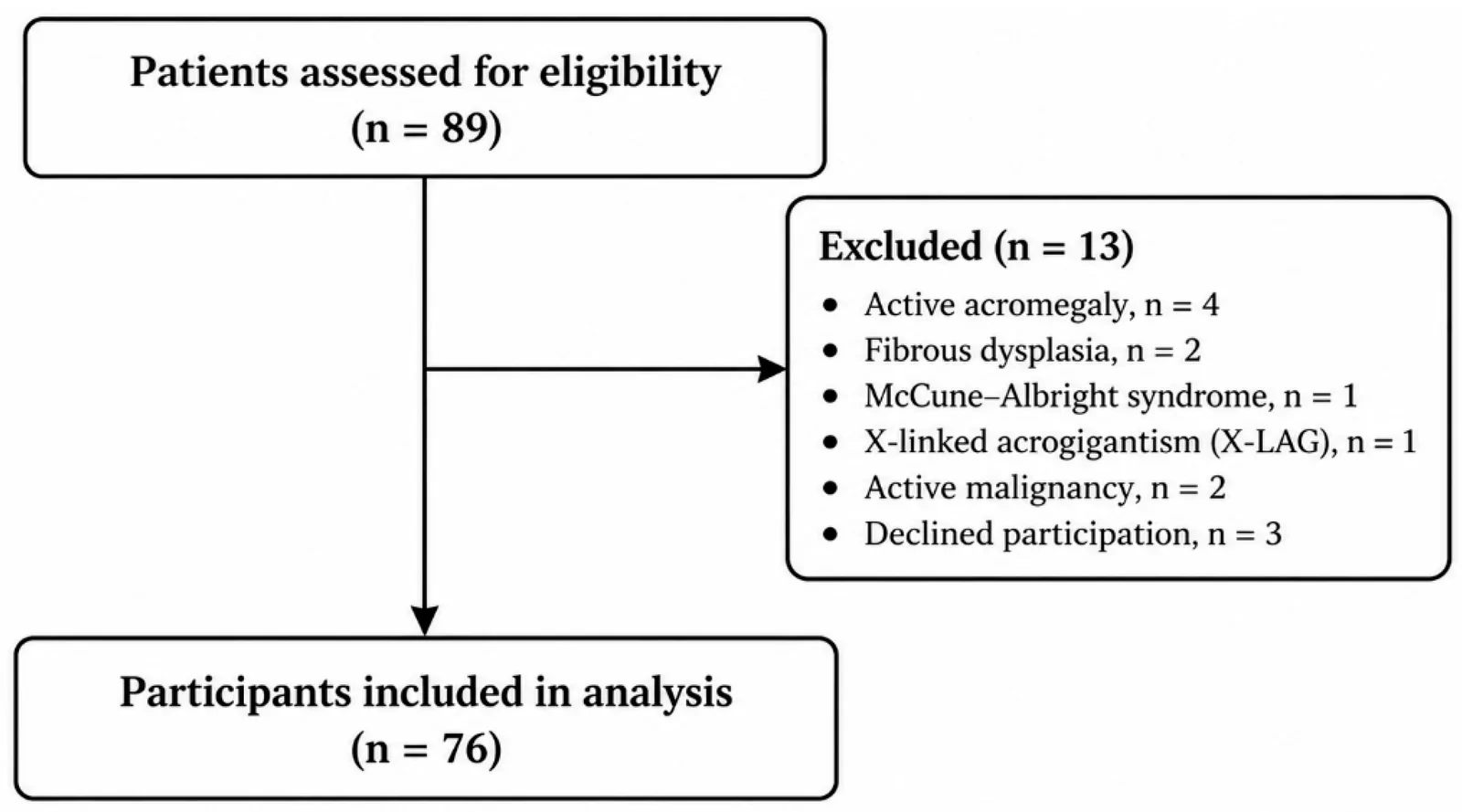
Participant flow diagram. Of 89 patients assessed for eligibility, 13 were excluded (4 with active acromegaly, 2 with fibrous dysplasia, 1 with McCune–Albright syndrome, 1 with X-linked acrogigantism, 2 with active malignancy, and 3 who declined participation), leaving 76 participants included in the analysis.

**Table 1:** Characteristics of the 76 participants with biochemically controlled acromegaly.

Characteristic	Overall Cohort (n = 76)
Age, years	48.8 ± 11.0
Female sex	38 (50.0)
Age at diagnosis, years	36.0 (30.0–44.2)
Follow-up, months	118.5 (81.8–158.8)
Duration of biochemical control, months	65.5 (36.0–113.2)
Post-presentation interval to sustained control, months	29.0 (15.8–70.0)
Baseline IGF-1 index	2.44 (1.71–3.27); n = 66
Current IGF-1 index	0.37 (0.28–0.50)
Pituitary surgery	66 (86.8)
Ever received medical therapy	53 (69.7)
Current medical therapy	33 (43.4)
Current somatostatin receptor ligand (SRL)	32 (42.1)
Octreotide	13 (17.1)
Lanreotide	19 (25.0)
Current dopamine agonist	22 (28.9)
Current pegvisomant	0 (0.0)
Ever received pegvisomant	4 (5.3)
Previous radiotherapy	14 (18.4)
Hypopituitarism	20 (26.3)
Diabetes mellitus	31 (40.8)
Obesity	30 (39.5)
BMI, kg/m^2^	28.6 (26.5–32.0)
Fat mass, kg	22.9 (18.7–31.5)
Body fat, %	29.6 (22.9–35.6)
Fat-free mass, kg	61.2 (47.9–72.0)
Estimated SMM, kg	34.67 (27.10–40.74)
Maximum handgrip strength, kg	40.25 (31.68–51.33)
Relative handgrip strength, kg/kg	1.182 (1.053–1.349)
Low absolute handgrip strength	3 (3.9)
Low SMM/BMI	8 (10.5)
Impaired gait/chair-stand threshold	4 (5.3)/3 (3.9)

Values are mean ± standard deviation, median (interquartile range), or n (%). Treatment categories were not mutually exclusive. No participant was receiving pasireotide at assessment. Low absolute handgrip strength and low SMM/BMI were reported for descriptive purposes only and were not used to define the primary performance-group comparisons. The maximum BMI value of 77.4 kg/m^2^ was verified against the source anthropometric records. BMI, body mass index; IGF-1, insulin-like growth factor 1; SMM, estimated skeletal muscle mass; SRL, somatostatin receptor ligand.

**Table 2:** Handgrip strength and estimated skeletal muscle mass according to threshold-defined performance status.

**Panel A. Gait**						
**Test and Measure**	**Impaired (n = 4)**	**Not Impaired (n = 72)**	**Rank-Biserial r**	**Hodges–Lehmann Δ** **(Unadjusted 95% CI)**	**Exact *p***	**Holm *p***
Maximum handgrip strength, kg	26.65 (25.40–26.98)	41.50 (32.42–51.73)	−0.878	−15.80 (−27.60 to −5.30)	0.0007	0.0035
Estimated SMM, kg	27.37 (26.74–28.70)	34.95 (27.24–40.88)	−0.483	−6.88 (−13.92 to 0.79)	0.1088	0.2175
Relative handgrip strength, kg/kg	0.918 (0.851–0.985)	1.189 (1.088–1.360)	−0.847	−0.292 (−0.477 to −0.127)	0.0015	0.0061
**Panel B. Chair Stand**						
**Test and Measure**	**Impaired (n = 3)**	**Not Impaired (n = 73)**	**Rank-Biserial r**	**Hodges–Lehmann Δ** **(Unadjusted 95% CI)**	**Exact *p***	**Holm *p***
Maximum handgrip strength, kg	26.80 (24.45–29.45)	41.10 (32.20–51.70)	−0.767	−13.80 (−29.10 to −1.40)	0.0185	0.0555
Estimated SMM, kg	31.41 (28.78–40.36)	34.70 (27.11–40.70)	0.018	0.45 (−10.58 to 14.60)	0.9624	0.9624
Relative handgrip strength, kg/kg	0.845 (0.748–0.849)	1.188 (1.073–1.357)	−0.973	−0.404 (−0.637 to −0.209)	0.0002	0.0012

Values are medians (interquartile ranges). A negative rank-biserial r indicates a lower value in the impaired group. Exact *p* values are from fully enumerated, tie-aware permutation tests. The Hodges–Lehmann Δ is the median of all pairwise differences (impaired minus not impaired); the corresponding 95% CIs are unadjusted and were obtained by inversion of the exact Mann–Whitney test. Holm adjustment was applied jointly across the six comparisons in both panels. The six full-cohort comparisons form the originally defined primary analysis; the results of the post hoc sex-stratified sensitivity analysis are reported in the text and Supplementary Table S1. SMM, estimated skeletal muscle mass.

**Table 3:** Complementary multivariable models of relative and absolute handgrip strength.

**Panel A. Outcome: Relative Handgrip Strength (kg/kg)**			
**Predictor**	**Unstandardized Coefficient (B)**	**95% CI**	***p***
Intercept	1.261	0.997 to 1.525	<0.001
Age, per 10 years	0.024	−0.033 to 0.081	0.417
Female sex	−0.133	−0.233 to −0.033	0.009
Obesity	−0.136	−0.242 to −0.030	0.012
Diabetes mellitus	−0.059	−0.163 to 0.045	0.265
Interval to sustained control, per 5 years	−0.049	−0.162 to 0.063	0.390
Previous radiotherapy	−0.059	−0.162 to 0.044	0.259
Current medical therapy	−0.005	−0.115 to 0.104	0.924
**Panel B. Outcome: Absolute Handgrip Strength (kg), with Estimated SMM as a Covariate**			
**Predictor**	**Unstandardized Coefficient (B)**	**95% CI**	***p***
Intercept	14.289	−15.528 to 44.107	0.348
Estimated SMM, per 5 kg	4.733	1.299 to 8.168	0.007
Age, per 10 years	0.304	−1.773 to 2.380	0.774
Female sex	−7.581	−16.786 to 1.623	0.106
Obesity	−4.280	−9.697 to 1.137	0.122
Diabetes mellitus	−2.444	−6.439 to 1.552	0.231
Interval to sustained control, per 5 years	−1.164	−4.791 to 2.463	0.529
Previous radiotherapy	−1.627	−5.249 to 1.996	0.379
Current medical therapy	−0.522	−5.014 to 3.970	0.820

(Panel A) n = 76; R^2^ = 0.290; adjusted R^2^ = 0.217. Model uses HC3 robust standard errors. Robust overall Wald F-test (all seven predictors = 0): F(7,68) = 3.30, *p* = 0.0044. R^2^ and adjusted R^2^ are conventional OLS measures and are not adjusted for the use of HC3 robust standard errors; n = 76; (Panel B) R^2^ = 0.728; adjusted R^2^ = 0.695. Model uses HC3 robust standard errors. Robust overall Wald F-test (all eight predictors = 0): F(8,67) = 22.12, *p* < 0.001. R^2^ and adjusted R^2^ are conventional OLS measures and are not adjusted for the use of HC3 robust standard errors. CI, confidence interval; SMM, estimated skeletal muscle mass.

## Data Availability

The datasets generated and/or analyzed during the current study are not publicly available because they contain patient-level clinical information governed by institutional ethics and privacy requirements, but deidentified data may be available from the corresponding author on reasonable request and subject to institutional approval.

## Supplementary Materials

The following supporting information can be downloaded at: https://www.mdpi.com/article/10.3390/jcm15197412/s1, Figure S1: Participant-level distributions of maximum handgrip strength, estimated skeletal muscle mass, and relative handgrip strength according to gait and chair-stand status; Figure S2: Scatterplot of maximum handgrip strength against estimated skeletal muscle mass, stratified by sex; Table S1: Post hoc women-only sensitivity analyses of threshold-defined performance status; Table S2: Leave-one-out sensitivity analysis of the full-cohort comparisons, and collinearity, influence, and residual diagnostics for the multivariable models; Table S3: Analyses addressing the clustering of impairment among women with diabetes, and characteristics of affected participants.

## Abbreviations

BMI, body mass index; CI, confidence interval; FFM, fat-free mass; GH, growth hormone; HC3, heteroskedasticity-consistent covariance estimator type 3; IGF-1, insulin-like growth factor 1; MRI, magnetic resonance imaging; SMM, estimated skeletal muscle mass; SRL, somatostatin receptor ligand.
