# Supplementary material for: Handgrip Strength Relative to Estimated Skeletal Muscle Mass and Threshold-Defined Physical Performance in Biochemically Controlled Acromegaly: A Cross-Sectional Study
Source: J Clin Med. 2026 Sep 24;15(19):7412. doi: 10.3390/jcm15197412 (PMC13666115; doi:10.3390/jcm15197412)
Supplement: Supplementary file 1 [file jcm-15-07412-s001.zip › jcm-4544567-supplementary.pdf]

Journal of
Clinical Medicine

MDPI

Article

Handgrip Strength Relative to Estimated Skeletal Muscle Mass and Threshold-Defined Physical Performance in Biochemically Controlled Acromegaly: A Cross-Sectional Study

Ayşe Merve Ok Kurt [1,2,*], Ümmü Mutlu [1], Fatih Bektaş [1,3], Hülya Hacişahinoğulları [1], Gülşah Yenidünya Yalın [1], Tuğba Erdoğan [4], Gulistan Bahat [4], Nurdan Gül [1], Ayşe Kubat Üzüm [1], Ferihan Aral [1], Sema Yarman [1] and Özlem Soyluk Selçukbiricik [1]

[1] Division of Endocrinology and Metabolism, Department of Internal Medicine, Istanbul Faculty of Medicine, Istanbul University, 34093 Istanbul, Türkiye; ukorkmaz18@gmail.com (Ü.M.); drfatihbektas@gmail.com (F.B.); mercandogru@hotmail.com (H.H.); gulsah_y@hotmail.com (G.Y.Y.); nurdan.gul@istanbul.edu.tr (N.G.); ayse.kubat@istanbul.edu.tr (A.K.Ü.); ferihanar@yahoo.com (F.A.); sema.yarman@gmail.com (S.Y.); drozlem76@hotmail.com (Ö.S.S.)
[2] Department of Endocrinology and Metabolism, Kocaeli City Hospital, 41060 Kocaeli, Türkiye
[3] Department of Endocrinology and Metabolism, Bağcılar Training and Research Hospital, 34200 Istanbul, Türkiye
[4] Division of Geriatrics, Department of Internal Medicine, Istanbul Faculty of Medicine, Istanbul University, 34093 Istanbul, Türkiye; tubaobekli@hotmail.com (T.E.); gbahatozturk@yahoo.com (G.B.)
* Correspondence: a.merve52@gmail.com

These supplementary materials present participant-level distributions of the primary measures (Figure S1), a sex-stratified scatterplot of handgrip strength against estimated SMM supporting the allometric sensitivity analysis (Figure S2), post hoc women-only sensitivity analyses (Table S1), leave-one-out sensitivity analyses and collinearity, influence, and residual diagnostics for the multivariable models (Table S2), and analyses addressing the clustering of impairment among women with diabetes together with characteristics of the affected participants (Table S3).

Interval denotes the post-presentation interval to sustained biochemical control and is not lifetime duration of active disease. BMI, body mass index; SMM, estimated skeletal muscle mass.

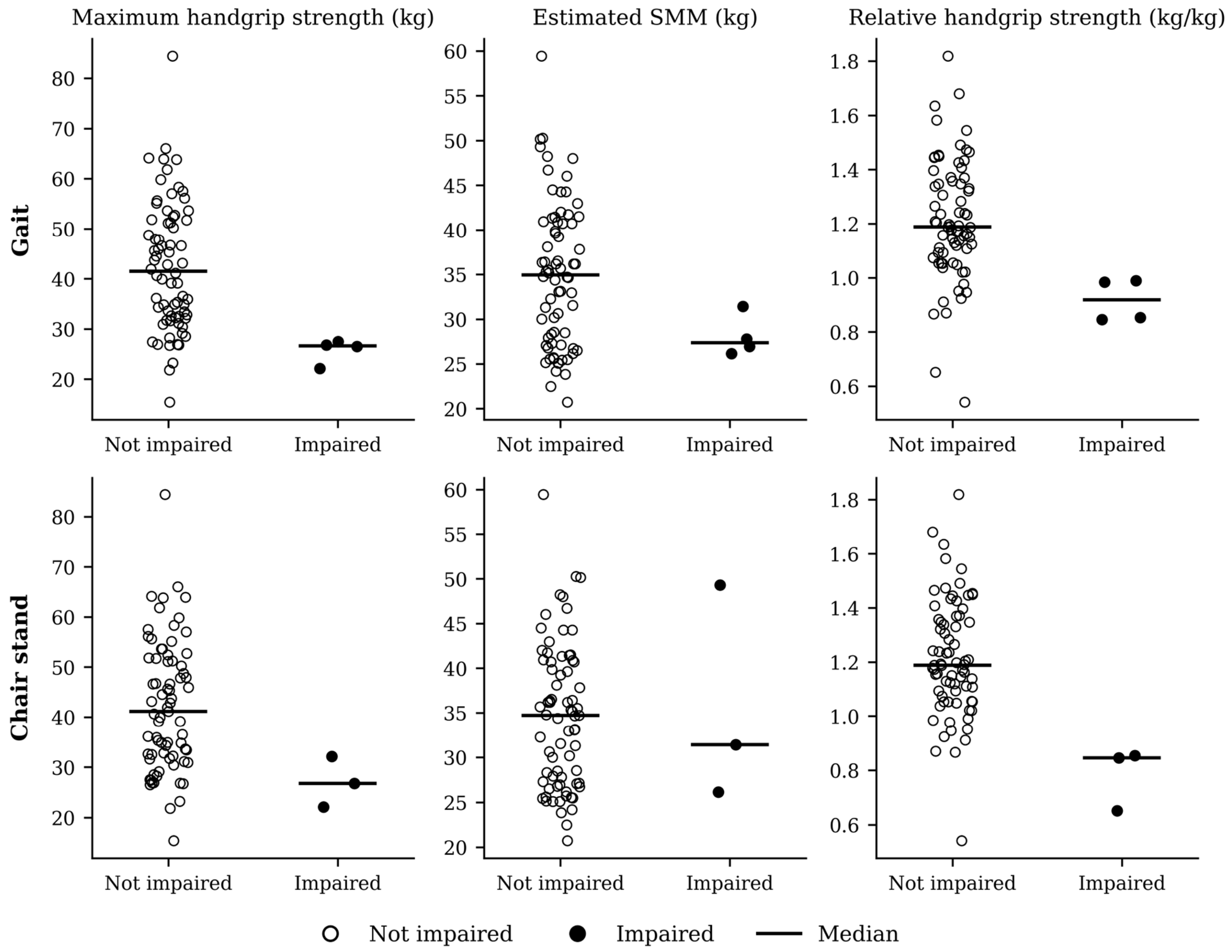

Figure S1. Participant-level distributions of maximum handgrip strength, estimated skeletal muscle mass (SMM), and relative handgrip strength (handgrip/SMM), grouped by gait status (top row) and chair-stand status (bottom row). Open circles denote participants who did not meet the corresponding impairment threshold; filled circles denote participants who met it. Horizontal bars indicate the group median. Points are jittered horizontally within each group to reduce overlap; the x-axis position carries no other meaning. Gait groups: impaired n = 4, not impaired n = 72. Chair-stand groups: impaired n = 3, not impaired n = 73.

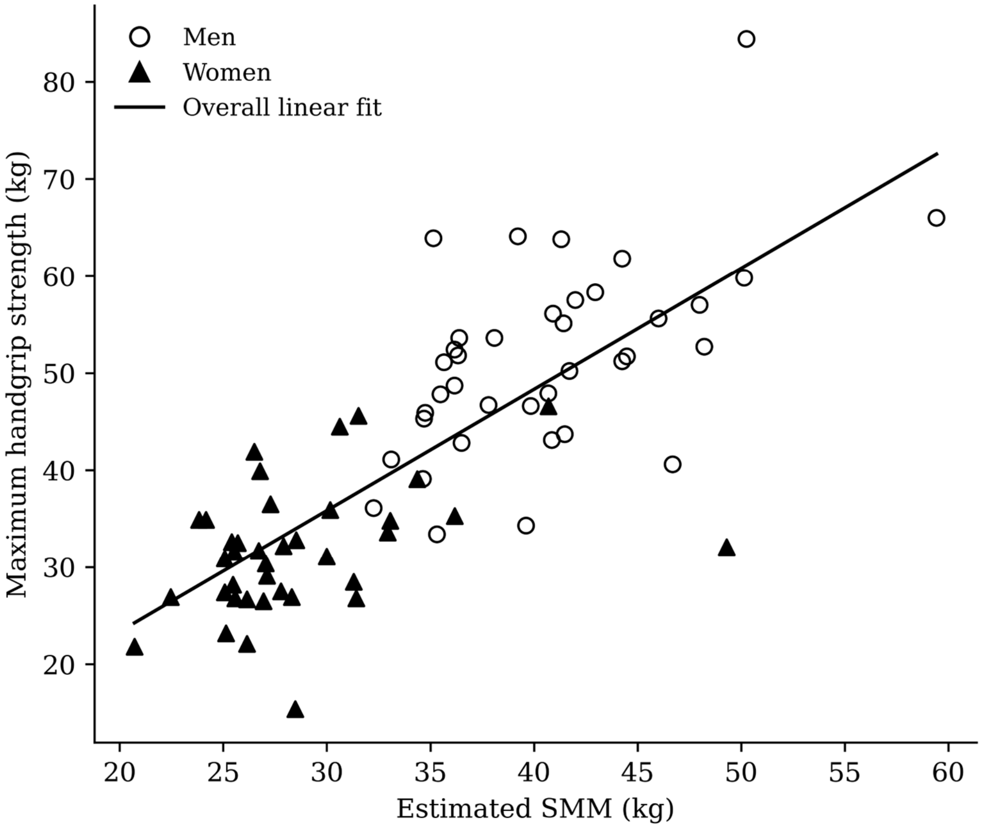

Figure S2. Scatterplot of maximum handgrip strength against estimated skeletal muscle mass (SMM), stratified by sex. Open circles denote men (n = 38); filled triangles denote women (n = 38). The solid line is the overall (unstratified) linear fit. This figure accompanies the allometric sensitivity analysis described in the main text: the log–log slope relating handgrip strength to estimated SMM was 1.073 (not different from 1, p = 0.49), the untransformed intercept did not differ from zero (p = 0.73), and there was no evidence of a sex interaction (p = 0.44).

Table S1. Post hoc women-only sensitivity analyses of threshold-defined performance status

Test and measure	Impaired	Not impaired	Rank-biserial r	Permutation p	Holm-adjusted p
Gait: Maximum handgrip strength, kg	26.65 (25.40–26.98)	32.15 (28.27–35.20)	-0.743	0.0122	0.0486
Gait: Estimated SMM, kg	27.37 (26.74–28.70)	27.08 (25.48–30.51)	0.096	0.7716	0.7716
Gait: Relative handgrip strength, kg/kg	0.918 (0.851–0.985)	1.142 (1.039–1.258)	-0.765	0.0097	0.0486
Chair stand: Maximum hand-grip strength, kg	26.80 (24.45–29.45)	31.70 (27.45–35.10)	-0.514	0.1545	0.4634
Chair stand: Estimated SMM, kg	31.41 (28.78–40.36)	27.05 (25.50–30.08)	0.457	0.2074	0.4634
Chair stand: Relative handgrip strength, kg/kg	0.845 (0.748–0.849)	1.138 (1.029–1.251)	-0.943	0.0017	0.0100

Gait groups: impaired n = 4 and not impaired n = 34. Chair-stand groups: impaired n = 3 and not impaired n = 35. Values are median (interquartile range). Permutation p values are exact, tie-aware, two-sided p values from the women-only comparisons; Holm-adjusted p values apply the Holm step-down correction across the six comparisons in this table. These analyses were added post hoc after all impaired participants were found to be women; they are sensitivity analyses of the six full-cohort comparisons in main-text Table 2, which remain the primary analysis. SMM, estimated skeletal muscle mass.

Table S2. Leave-one-out sensitivity analysis of the full-cohort comparisons, and collinearity, influence, and residual diagnostics for the multivariable models

Panel A. Leave-one-out analysis of the gait comparisons (Holm-adjusted p, six-comparison family)

Participant excluded	Maximum handgrip	Relative handgrip	Estimated SMM
None (original, n = 4 vs. 72)	0.0035	0.0061	0.2175
P1 (72 y, BMI 28.6 kg/m²)	0.0237	0.0209	0.4070
P3 (47 y, BMI 43.9 kg/m²)	0.0206	**0.0521**	0.2050
P4 (71 y, BMI 27.7 kg/m²)	0.0400	**0.0585**	0.5047
P5 (62 y, BMI 35.9 kg/m²)	0.0078	0.0140	0.3349

Each row shows the Holm-adjusted p value for the six-comparison family after removing one gait-impaired participant (leaving n = 3 vs. 72) while holding the chair-stand comparisons at their original values. Exact permutation p values were calculated using tie-aware mid-ranks; Holm adjustment was reapplied to the six comparisons within each leave-one-out scenario. Bold values exceed p = 0.05. P1, P3, P4, and P5 denote the participants described in Table S3, Panel C; P2 is not listed because that participant met only the chair-stand threshold. SMM, estimated skeletal muscle mass.

Panel B. Leave-one-out analysis of the chair-stand comparisons (Holm-adjusted p, six-comparison family)

Participant excluded	Maximum handgrip	Relative handgrip	Estimated SMM
None (original, n = 3 vs. 73)	0.0555	0.0012	0.9624
P2 (41 y, BMI 77.4 kg/m²)	**0.0442**	0.0253	0.3256
P3 (47 y, BMI 43.9 kg/m²)	0.2747	0.0112	0.7667
P4 (71 y, BMI 27.7 kg/m²)	0.3263	0.0168	0.4204

Each row shows the Holm-adjusted p value for the six-comparison family after removing one chair-stand–impaired participant (leaving n = 2 vs. 73) while holding the gait comparisons at their original values. Exact permutation p values were calculated using tie-aware mid-ranks; Holm adjustment was reapplied to the six comparisons within each leave-one-out scenario. Bold indicates the sole removal scenario in which the maximum-handgrip comparison, non-significant in the full three-participant group, fell below p = 0.05; it remained above 0.05 in the other two removal scenarios. P2, P3, and P4 denote the participants described in Table S3, Panel C; P1 and P5 are not listed because they met only the gait threshold. SMM, estimated skeletal muscle mass.

Panel C. Variance inflation factors for the multivariable models in main-text Table 3

Predictor	Model A (relative handgrip)	Model B (absolute handgrip)
Estimated SMM, per 5 kg	—	3.78
Age, per 10 years	1.50	1.75
Female sex	1.15	3.06
Obesity	1.05	1.52
Diabetes mellitus	1.31	1.32
Interval to sustained control, per 5 years	1.80	1.80
Previous radiotherapy	1.26	1.26
Current medical therapy	1.21	1.21

All variance inflation factors were below conventional thresholds for concern (commonly 5–10) in both models. SMM, estimated skeletal muscle mass.

Panel D. Influence diagnostics (Cook's distance) for the multivariable models in main-text Table 3

Participant (BMI)	Cook's D (Model A)	Cook's D (Model B)	Exceeds 4/n threshold[1]	Excluded in BMI-based sensitivity analysis
BMI 23.2 kg/m² (not obese, diabetic)	0.190	0.095	Model A and Model B	No
BMI 27.7 kg/m² (P4, impaired group)	0.072	—	Model A	No
BMI 33.4 kg/m²	0.071	—	Model A	No
BMI 33.1 kg/m²	0.061	—	Model A	No
BMI 77.4 kg/m² (P2, impaired group)	0.057	0.326	Model A and Model B	Yes
BMI 26.0 kg/m² (young, male, non-diabetic)	—	0.214	Model B	No

[1] Threshold = 4/n = 0.053. Only observations exceeding the threshold in either model are listed; dashes indicate the observation did not exceed the threshold in that model. The participant with BMI 77.4 kg/m² was the most influential observation for Model B (absolute handgrip strength) but was not the most influential observation for Model A (relative handgrip strength), where a different, non-obese participant with diabetes had a higher Cook's distance. P2 and P4 denote the participants labeled P2 and P4 in Table S3, Panel C.

Sensitivity analyses excluding influential observations showed that the relative-handgrip model was generally stable: the coefficients for female sex and obesity remained negative and below the 0.05 threshold after excluding the participant with BMI 77.4 kg/m², the observation with the highest Cook's distance, or both. In the absolute-handgrip model, the sex and obesity coefficients were more sensitive to which influential observation was excluded, whereas the positive coefficient for estimated SMM was retained.

Panel E. Residual diagnostics and linearity checks for the multivariable models in main-text Table 3

Diagnostic	Model A (relative handgrip)	Model B (absolute handgrip)
Breusch–Pagan test for heteroscedasticity, p	0.612	0.227
Shapiro–Wilk test for residual normality, p	0.330	0.510
Ramsey RESET test for functional form, p	0.714	0.093
Linearity of age (squared-term p)	0.249	0.717
Linearity of interval to sustained control (squared-term p)	0.905	0.917
Linearity of estimated SMM (squared-term p)	—	0.795

Diagnostics were computed on the classical (non-robust) ordinary least squares fit of each model (n = 76) underlying the HC3-robust estimates reported in main-text Table 3. The Breusch–Pagan test provides a formal check supporting the use of HC3 robust standard errors regardless of its result. Linearity of each continuous predictor was assessed by adding its squared term to the model and testing its coefficient; none reached conventional significance, providing no evidence against the linearity assumption. SMM, estimated skeletal muscle mass.

Table S3. Analyses addressing the clustering of impairment among women with diabetes, and characteristics of affected participants

Panel A. Exploratory analysis: six comparisons restricted to women with diabetes (n = 21)

Test and measure	Impaired	Not impaired	Rank-biserial r	Exact p	Holm p
Gait: Maximum handgrip strength, kg	26.65 (25.40–26.98)	32.10 (29.10–34.90)	-0.794	0.0120	0.0632
Gait: Estimated SMM, kg	27.37 (26.74–28.70)	26.77 (25.41–30.00)	0.265	0.4618	0.4618
Gait: Relative handgrip strength, kg/kg	0.92 (0.85–0.99)	1.19 (0.98–1.28)	-0.588	0.0805	0.3221
Chair stand: Maximum handgrip strength, kg	26.80 (24.45–29.45)	31.35 (28.27–34.90)	-0.481	0.2165	0.4331
Chair stand: Estimated SMM, kg	31.41 (28.78–40.36)	26.86 (25.43–28.30)	0.593	0.1248	0.3744
Chair stand: Relative handgrip strength, kg/kg	0.85 (0.75–0.85)	1.15 (0.99–1.28)	-0.889	0.0105	0.0632

Gait groups: impaired n = 4 and not impaired n = 17. Chair-stand groups: impaired n = 3 and not impaired n = 18. Values are median (interquartile range). Exact p values are tie-aware, two-sided permutation p values. A negative rank-biserial r indicates a lower value in the impaired group, consistent with the sign convention used in Table 2. Holm adjustment was applied across the same six-comparison family used for the full-cohort analysis. SMM, estimated skeletal muscle mass.

Panel B. Post hoc sex-stratified sensitivity analysis compared with the women-only analysis

Test and measure	Exact stratified permutation p	Van Elteren Z (asymptotic)	Van Elteren p (asymptotic)	Women-only exact p (Table S1)
Gait: Maximum handgrip strength	0.0122	-2.402	0.0163	0.0122
Gait: Relative handgrip strength	0.0097	-2.473	0.0134	0.0097
Gait: Estimated SMM	0.7716	0.309	0.7572	0.7716
Chair stand: Maximum handgrip strength	0.1545	-1.462	0.1438	0.1545
Chair stand: Relative handgrip strength	0.0017	-2.680	0.0074	0.0017
Chair stand: Estimated SMM	0.2074	1.299	0.1938	0.2074

An exact permutation test stratified by sex enumerates all relabelings of the impairment indicator that preserve the observed number of impaired participants within each sex stratum. Because zero of the 38 men met either threshold, there is only one possible allocation within the male stratum, so every valid stratified permutation differs from the others only in which women are labeled as impaired; the exact stratified permutation p value is therefore identical to the exact women-only permutation p value in Table S1, which was confirmed by full enumeration for all six comparisons. As a secondary, model-based check, an asymptotic Van Elteren (stratified Wilcoxon) statistic yielded closely concordant results. The sex-stratified analysis was added post hoc after all impaired participants were found to be women; it is a sensitivity analysis of the six full-cohort comparisons and does not address the concurrent clustering of impairment among participants with diabetes. Holm-adjusted p values just below 0.05 in this analysis should not be read as stronger evidence than the full-cohort analysis.

Panel C. Characteristics of the five participants meeting an impairment threshold

Characteristic	P1	P2	P3	P4	P5	⚜nimpaired women (n = 33)
Threshold met	Gait	Chair stand	Gait + chair stand	Gait + chair stand	Gait	—
Age, years	72	41	47	71	62	49 (42–57)

Characteristic	P1	P2	P3	P4	P5	⚜nimpaired women (n = 33)
BMI, kg/m^2	28.6	77.4	43.9	27.7	35.9	28.6 (26.1–33.4)
Obesity	No	Yes	Yes	No	Yes	13 (39.4%)
Estimated SMM, kg	26.9	49.3	31.4	26.1	27.8	27.1 (25.5–30.2)
Maximum handgrip strength, kg	26.5	32.1	26.8	22.1	27.5	32.2 (28.2–35.3)
Relative handgrip strength, kg/kg	0.98	0.65	0.85	0.85	0.99	1.15 (1.05–1.26)
Meets low absolute handgrip threshold	No	No	No	No	No	3 (9.1%)
Current acromegaly treatment	None	SRL	SRL + DA	SRL	SRL + DA	13 (39.4%) on therapy
Current IGF-1 index	0.38	0.73	0.95	0.49	0.32	0.35 (0.24–0.49)
Interval to sustained control, months	112	22	154	173	80	20 (9–36)
Pituitary surgery	Yes	No	Yes	Yes	No	32 (97.0%)

P1–P5 denote the five participants meeting a gait and/or chair-stand impairment threshold; all were women with diabetes. The same labels are used in Table S2, Panels A, B, and D. Values for unimpaired women are median (interquartile range) or n (%). The low absolute handgrip strength threshold (<20 kg in women) is the same descriptive threshold reported in Table 1; none of the five participants met it despite meeting the physical-performance threshold, and none of the three participants in the overall cohort who met it had impaired gait or chair-stand performance. SRL, somatostatin receptor ligand; DA, dopamine agonist; BMI, body mass index; SMM, estimated skeletal muscle mass.
